# Functional and Neural Correlates Associated with Conditioned Pain Modulation in Patients with Chronic Knee Osteoarthritis Pain: A Cross-Sectional Study

**DOI:** 10.3390/life13081697

**Published:** 2023-08-07

**Authors:** Marcel Simis, Kevin Pacheco-Barrios, Karen Vasquez-Avila, Ingrid Rebello-Sanchez, Joao Parente, Luis Castelo-Branco, Anna Marduy, Paulo S. de Melo, Marta Imamura, Linamara Battistella, Felipe Fregni

**Affiliations:** 1 Faculdade de Medicina, Hospital das Clínicas HCFMUSP, Universidade de São Paulo, São Paulo 01002, Brazil; marcel.simis@hc.fm.usp.br (M.S.); marta.imamura@fm.usp.br (M.I.); linamara.battistella@hc.fm.usp.br (L.B.); 2 Neuromodulation Center and Center for Clinical Research Learning, Spaulding Rehabilitation Hospital and Massachusetts General Hospital, Harvard Medical School, Boston, MA 02129, USA; kevin.pacheco.barrios@gmail.com (K.P.-B.); kvasquez2705@gmail.com (K.V.-A.); ingridrsnunes@gmail.com (I.R.-S.); j.v.parente1997@gmail.com (J.P.); leccbranco@gmail.com (L.C.-B.); anna.marduy@gmail.com (A.M.); melosrpaulo@gmail.com (P.S.d.M.); 3 Unidad de Investigación para la Generación y Síntesis de Evidencia en Salud, Universidad San Ignacio de Loyola, Vicerrectorado de Investigación, Lima 15026, Peru

**Keywords:** conditioned pain modulation, osteoarthritis, pain, race, balance

## Abstract

**Background:** In this study, we aimed to assess the factors that predict a dysfunctional conditioned pain modulation (CPM) in chronic knee OA. **Methods:** This is a cross-sectional analysis of patients with chronic knee OA from a prospective cohort study in Brazil (n = 85). We performed linear and logistic multivariate regression models using the purposeful selection approach to test the relationship between the CPM in both knees (average) as a dependent variable and demographics, clinical, and neurophysiological as independent variables. **Results:** A significant negative association between WOMAC pain scores and CPM (β: −0.13) was found. This association was modified by the subjects’ race, being stronger in the non-white subjects. In our logistic regression models, pain intensity indexed with the WOMAC pain scale remained a significant association with dichotomized CPM. Furthermore, a significant CPM association with balance, indexed with the Berg Balance score, was evidenced (β: 0.04). Neurophysiological variables showed a significant negative relationship with CPM, such as the relative power of delta oscillations in the frontal area (β: −3.11) and central area (β: −3.23). There was no significant relationship between CPM and the following domains: cognitive, emotion, sleep, opioid receptor polymorphisms, and intrinsic variables of OA disease. There was no association of CPM with TMS-indexed inhibitory markers. **Conclusions:** These results may indicate that less function of the pain descending inhibitory system in patients with OA is correlated with higher activity-related pain (WOMAC), less balance, and cortical plasticity especially with increased low-frequency (delta) brain oscillations. These associations seem modified by race.

## 1. Introduction

Osteoarthritis (OA) is a chronic progressive degenerative joint disease characterized by pain, stiffness, and movement restriction [1]. The most common site of OA is the knee joint, and OA is one of the leading causes of disability and chronic pain in the elderly population [1,2]. The underlying mechanism of pain in OA is yet to be elucidated. The localized inflammation process is a core event; however, it is also believed to be accompanied by a state of hypersensitivity involving cascades of peripheral and central sensitization as well as modulation by descending pathways [3,4,5].

Studies have investigated how these mechanisms are implicated in the chronic pain experience in OA. Although there is no gold standard measure of pain sensitization in humans, Quantitative Sensory Testing (QST) is the most frequently used paradigm to study nociception [6]. Conditioned Pain Modulation (CPM) is an experimental dynamic QST measure that is thought to reflect the activity of the endogenous pain modulation system–a supraspinal top-down modulation of pain processing. Under certain experimental settings, it mediates the phenomena of “pain inhibits pain”. The experiment typically consists of an evaluation of the change in the response to a painful test stimulus before and after the delivery of a conditioning noxious stimuli. Most healthy individuals report a reduction in nociception [7,8]. Therefore, as an emerging pain biomarker, it needs more research to validate and better understand how CPM works and its relationships with other different variables [9].

The impairment of this mechanism (i.e., no changes or augmented pain perception after a conditioning stimulus) is suggested to be a key factor in the development and maintenance of chronic musculoskeletal pain syndromes [6,10].

Dysfunctional CPM is a replicated finding in OA pain studies [11,12,13,14]. However, CPM is not homogeneously affected across different KOA samples and strata, and the clinical, functional, and neural correlates of CPM status well established [14,15,16,17]. Therefore, CPM presents itself as a promising tool that could be used to assess the pain phenotype of subjects with OA [18] and create individualized tailored pain treatment. To accomplish this, it is crucial to acquire a better understanding of the CPM paradigm, including the factors that affect it. For this reason, the objective of our study is to assess the factors that predict a dysfunctional CPM in our sample of chronic knee OA. Based on previous literature, we hypothesize that the female sex, older age, black race, the presence of theta and delta waves, higher short intracortical inhibition (SICI) conveyed by transcranial magnetic stimulation (TMS), and higher pain catastrophizing and pain scores would contribute to an impaired CPM [19,20,21,22,23].

## 2. Materials and Methods

### 2.1. Study Design and Participants

This is a cross-sectional analysis of patients with chronic knee OA (n = 85) from a prospective cohort study “Deficit of Inhibition as a Marker of Neuroplasticity (DEFINE study) in rehabilitation” [24] in São Paulo, Brazil. This study was approved by the Research and Ethical Committee of Hospital das Clínicas da Faculdade de Medicina da Universidade de São Paulo (HC FMUSP) (Registration number: 86832518.7.0000.0068) and follows the Brazilian research ethics regulations and the Declaration of Helsinki. For further information regarding the inclusion/exclusion criteria, see Text S1 in Supplementary material.

The study sample consisted of 85 subjects from the Instituto de Medicina Física e Reabilitação (IMREA) during the year 2019. The participants included fulfilled the following inclusion criteria: (1) be older than 18 years of age; (2) be male or female; (3) have a clinical and radiological (magnetic resonance imaging or computerized tomography, or bilateral knee radiography) diagnosis of knee osteoarthritis; (4) have clinical stability verified by medical evaluation; (5) have signed the informed consent to participate in the study personally or by his/her legal representative; and (6) meet the eligibility criteria for the IMREA rehabilitation program [24].

The exclusion criteria were the following: (1) pregnant women; (2) have an active osteoarthrosis with clinical manifestations in joints different than the knee; and (3) have any other clinical and/or social conditions that make it difficult for the subject to participate in the rehabilitation treatment.

### 2.2. Assessments

In the baseline visit, a trained physician performed and collected data from the participants regarding demographic variables and clinical, functional, and neurophysiological assessments.

### 2.3. Demographic Variables

We collected the participant’s information about their sex, age, race, educational level, weight, height, and body mass index. Race included white, black, mixed, and Asians. For the analysis of race, we decided to create two groups: the first group is the black race group that includes the black and mixed-race participants, and the second group is the non-black race group that includes the white and Asian participants.

### 2.4. Pain-Related Variables

We used different scales for the assessment of pain: the visual analog scale for pain (VAS Pain), the 36-item short form (SF-36), and the Western Ontario and McMaster Universities Osteoarthritis Index (WOMAC) pain scale.

Moreover, we used quantitative sensory testing (QST) for experimental pain assessment that included the following:

Pressure Pain Threshold (PPT) consists of a test where pressure is applied to a pre-specified region (thenar and above the knee) using an algometer with the objective to identify the minimum amount of pressure that triggers pain [25]. Three measurements with an interval of 15 s between them were made, and the average was calculated.

Conditioned pain modulation (CPM): We used a CPM protocol based on changes in PPTs [26,27].

Participants were asked to immerse one hand in a cold-water recipient (10–12 °C) for 1 min. After 30 s of immersion, the investigator showed VAS Pain so the subject could identify his/her level of pain related to the submerged hand. Then, three algometry measures (with intervals of 15 s) on the contralateral hand were taken, and then, the average of these measurements was recorded. After an interval of 10 min (time for the body temperature to return to normal) the other hand was immersed in the cold water and followed the same protocol aforementioned [27]. The CPM response was calculated using the difference between the average PPTs minus the average PPTs during the conditioned stimulus. Positive values were related to an effective CPM, and negative values were associated with an ineffective CPM. Moreover, to assess for clinical significance, we categorized our results in effective CPM if there was a change greater than 10% and ineffective CPM if there was a change lower than 10% [28,29].

### 2.5. Clinical Scales

The clinical domains of emotion, cognition, catastrophizing, sleep, motor function, balance, and functionality were measured with clinical standardized scales. In order to assess emotion, the Hamilton Depression Rating Scale (HAM-D) and the Hospital Anxiety and Depression Scale (HADS) were used. For catastrophizing, we used the Pain Catastrophizing scale and, for cognition, the Montreal Cognitive assessment (MOCA). In order to assess sleep and general functionality, we performed the Epworth sleepiness scale and the Medical Outcomes Short-Form Health Survey (SF-36), respectively. Moreover, to assess motor function, we used the Berg Balance Scale, the 10 m walking test, the 6 min walking test, and the Time Up and Go (TUG) test. In this study, we also aimed to assess the disease severity using the Kellgren–Lawrences Radiographic Classification of OA.

### 2.6. Genetic Variables

We assessed the genetic polymorphisms of the ABO system gene (rs505922), two polymorphisms of the OPRM1 gene (rs1799971 and rs1799972) and their possible relationship with pain, and a polymorphism of the BDNF gene (rs6265) [30].

### 2.7. Neurophysiological Variables

#### 2.7.1. Transcranial Magnetic Stimulation (TMS)

In our study, we employed the Magstim Rapid^®^ stimulator (The Magstim Company Limited, Whitland, UK) to evaluate TMS measurements. The resting motor threshold (rMT) was defined as the minimum intensity required for a single TMS pulse at the hot spot to produce a motor evoked potential (MEP) with a peak-to-peak amplitude of at least 50 μV in 5 out of 10 attempts [31]. We conducted various assessments, including MEP (at 120% of rMT with peak-to-peak amplitude calculation) and cortical silent period (CSP), which indicates the temporary suppression of electromyographic activity during sustained voluntary contraction. Additionally, we conducted paired-pulse protocols to assess intracortical inhibition (SICI) using interstimulus intervals of 2 ms and intracortical facilitation (ICF) using 10 ms interim stimulus intervals [31]. We applied ten randomized stimuli at each interval and calculated the averages. To obtain a bi-hemispheric average, we pooled the results of rMT, CSP, SICI, ICF, and MEP from both hemispheres. This approach is justified considering the bi-hemispheric nature of pain perception [32].

#### 2.7.2. Resting-State Electroencephalography (EEG)

For the resting-state EEG recordings, we followed a standardized procedure in a quiet room [33]. The participants were instructed to sit comfortably, gaze naturally below the horizon, refrain from moving or talking, and relax as much as possible. The investigator ensured that the participants did not fall asleep by observing them and alerting them verbally if drowsiness was detected. We recorded the resting-state EEG for a duration of 5 min with the participants’ eyes closed using a 128-channel EGI system (Electrical Geodesics, Inc.) based in Eugene, OR, USA. We calculated absolute power (μV2) and relative power (power within a specific frequency range divided by the total power from 1 to 40 Hz) for various frequency bands: delta (1–4 Hz), theta (4–8 Hz), alpha (8–13 Hz), beta (13–30 Hz), and two sub-bands that were low beta (13–20 Hz) and high beta (20–30 Hz). To focus on key cortical regions relevant to pain perception, namely the central, parietal, and frontal areas, we selected specific electrodes representing these regions and averaged the EEG measurements accordingly [34].

For more comprehensive details on the questionnaires and assessments used in our study, please refer to Text S1 in Supplementary material.

### 2.8. Statistical Analysis

Descriptive variables were reported as the mean and standard deviation if normal distributed and the median and interquartile range if non-non normal distributed. Variable distributions were assessed through histograms, q-q plot, kurtosis, and skewness.

**Figure 1:**
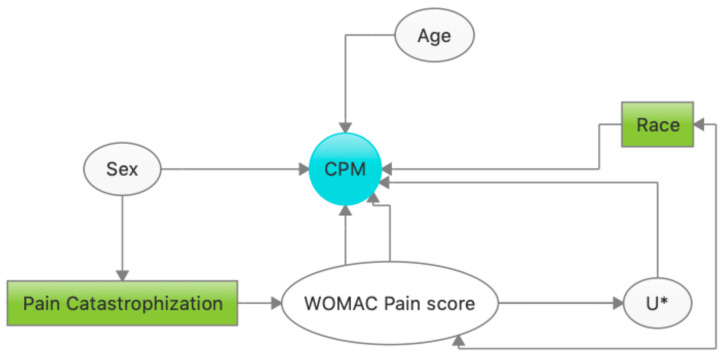
Directed Acyclic Graph of CPM. It illustrates the most common variables found in the literature associated with CPM and their pathways. The pathways that have a variable inside a square (green) are considered to be closed. U* refers to unmeasured variables in our study sample. In this case, it refers to drugs that subjects take due to pain.

Prior to the modeling stage, we searched for known factors that have been related to CPM in previous studies [19,20,21,22,23] and built a Directed Acyclic Graph (DAG) [35] to identify possible confounders to include in our model (Figure 1). The variables Pain Catastrophizing and Race were selected to block the backdoor pathways and, therefore, were adjusted to reduce confounding. Based on our DAG, we forced age and sex into the models due to literature suggesting that they are important biological confounders of CPM. Additionally, we tested effect modification by age and sex including product terms.

We aimed to model the relationship between the CPM in both knees (average) as a dependent variable, since most of the participants presented bilateral symptoms as continuous and categorical. For CPM categorization, we used two approaches: a recently published minimal clinically important difference on CPM for patients with OA [29] (1 = analgesia of 10% of more during CPM, 0 = analgesia of less than 10% during CPM) and a cut-off of zero change on CPM (1 = analgesia present, 0 = no analgesia (zero changes) or facilitation). Demographics, clinical, and neurophysiological variables were included as independent variables.

First, we performed linear and logistic univariate analyses to identify the covariates that would be included in our multivariate analysis. We used a forward approach for the variables selection. Variables with a *p* < 0.25 were selected (Tables in Supplementary material). We used the purposeful selection approach [36] or the variables selection process for building the model. We started with variables that had *p* < 0.05 from each one of the domains (demographics, pain-related, functional, general functionality, emotion, sleep, genetics, intrinsic variables of the disease, cognition, and neurophysiologic). Variables were added if they were significant, improved the adjusted R2, and/or were identified as confounders. Confounders were defined as variables that changed the B coefficient by more than 10% when compared to the previous model. The linearity and homoscedasticity assumptions were tested via graphical assessment. During the modeling stage, we assessed for multicollinearity between the variables and found that age and pain catastrophizing were collinear with the motor function variables (indexed as 10 min gait test, Berg Balance Scale [BBS]); thus, we did not include both in the models to maintain the additivity assumption in our models. The sample size calculation is reported in the protocol paper of the original cohort [24], which was estimated to be 100 participants under a very conservative scenario (99% power and alpha of 1%). In this cross-sectional analysis (85 included subjects), we had 84% power based on our previous calculation. The statistical analysis was performed using Stata Statistical Software 14 (Stata Corp LLC, College Station, TX, USA).

## 3. Results

### 3.1. Demographics and Characteristics of the Population

Our study sample consisted of 85 subjects with chronic knee OA (82% women and 15% men) aged 50 to 99 years (mean 68.46 years, SD 9.84 years); a total of 33% were black race (Black and mixed), and 67% were non-black (White and Asian). At baseline, the majority of patients had symptoms involving both knees and presented moderate pain. Also, they had low depression, anxiety, and cognitive scores. A detailed description of the sample size can be found in Table 1.

### 3.2. Pain-Related Variables and CPM

**Figure 2:**
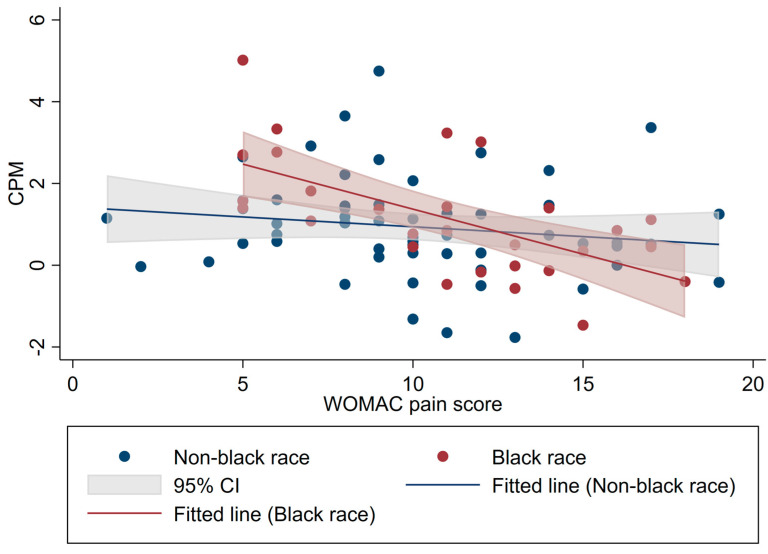
Effect modification by race.

In our first linear multivariate model for pain related variables (Table 2), we found a significant negative correlation between WOMAC pain scores and CPM (β: −0.13; 95%CI −0.21 to −0.04; *p* = 0.003), showing that subjects with higher WOMAC pain scores have a less effective CPM response. Moreover, this association can be modified by race; for instance, the magnitude of the association was stronger in the black race group, becoming more negative (Figure 2) compared to their non-black counterparts (interaction WOMAC*race [β: −0.15; 95%CI −0.29 to −0.01; *p* = 0.031]) (Table S3).

In the logistic multivariate models used to assess for clinical significance of CPM, we found that, when using the cut-off for CPM utilized by Tavares et al., there was a significant indirect correlation between WOMAC pain scores and CPM (OR: 0.79; 95%CI 0.67 to 0.94; *p* = 0.008), and VAS Pain bilateral also became significant (OR: 0.73, 95%CI 0.56 to 0.96, *p* = 0.022). In addition, when we assessed the relationships using the zero cut-off for CPM, there was a significant negative relationship between CPM and WOMAC Pain scores (OR: 0.73; 95%CI 0.58 to 0.91; *p* = 0.007) and also for VAS Pain bilateral (OR: 0.68; 95%CI: 0.49 to 0.95; *p* = 0.024) (See Tables in Supplementary material).

### 3.3. Functional Variables and CPM

As some motor function variables, such as the 6- and 10-min walking tests, were found to be collinear with age and pain catastrophizing, a different model (Table 3) was built that removed them to assess this domain. We found that subjects with higher scores in the Berg Balance Scale (BBS) had a direct relationship with CPM (B: −0.08; −0.156 to −0.018; *p* = 0.030). Moreover, the negative relationship between WOMAC Pain scores and CPM remained significant (β: 0.04; 95%CI: 0.003 to 0.080; *p* = 0.032).

### 3.4. Neurophysiological Variables and CPM

The multivariate analysis for brain oscillations and CPM showed that the relative power of delta waves in the frontal area has a strong negative relationship with CPM (β: −3.11; 95%CI: −5.90 to −0.33; *p* = 0.021) as well as in the central area (β: −3.23; 95%CI: −6.34 to −0.11; *p* = 0.040) (Table 4). There was no significant relationship between CPM and TMS variables.

### 3.5. Other Domains

There was no significant relationship between CPM and the following domains: cognitive, emotion, sleep, genetics, and intrinsic variables of the disease.

## 4. Discussion

Our results showed higher activity-related pain scores (WOMAC) associated with less effective CPM, and the magnitude of the association was larger if the subject was black or mixed race when compared to the other ethnic groups. Moreover, subjects with lower balance scores had a less effective CPM. Regarding neurophysiological parameters, a higher relative power of delta waves in the frontal and central areas was associated with a decrease in the effectiveness of CPM. We did not find a significant relationship between CPM and variables related to cognition, emotion, sleep, opioid receptor polymorphisms, TMS, or intrinsic characteristics of the disease.

### 4.1. Activity-Related Pain Associated with CPM

In the context of pain and CPM, an interesting paradigm was observed: while pain intensity indexed with the WOMAC scale remained significantly associated with CPM, pain intensity measured with VAS was only significant in our logistic regression models. It would make sense that a scale evaluating pain during daily activities would explain the course of pain and its magnitude [37]. In fact, we believe that a higher activation of the sensorimotor cortex would lead to a larger modulation of the pain and thus a better descending pain inhibitory system (DPMS) [25,38]. It is also possible that lower pain levels lead to more activity or a combination of both. This would ultimately lead to better scores on a balance test indexed with the BBS and lower WOMAC pain scores. In fact, some studies have suggested that VAS and the WOMAC scale measure different pain dimensions, those being a more general emotional dimension and a more sensory-mediated dimension, respectively [39,40]. Another one of our studies has shown emotional variables to predict pain indexed with VAS and functionality variables predicting pain measured through the WOMAC scale [41]. CPM is thought to be associated with the sensory-discriminative dimension of pain, considering it relates more to the primary and secondary somatosensory cortices and spinothalamic aspects of the DPMS, thus reflecting more functional aspects of pain [42]. CPM is possibly impacted by affective/motivational and non-specific pain pathways as it also interacts with areas such as the periaqueductal gray (PAG) [42]. Our results convey, therefore, the WOMAC pain scale as a more robust outcome for pain associated with CPM in osteoarthritis as it remained significant even when CPM was categorized.

### 4.2. Race as a Socioeconomic Surrogate

The relationship between pain indexed with the WOMAC and CPM was mediated by race in one of our models. Black and mixed-race individuals had more pain and less effective CPM in comparison to their non-black counterparts. Race could be functioning as a surrogate for socioeconomic status, considering the average income of black individuals is half compared to white people in Brazil [43]. In fact, studies in chronic pain convey a lack of significant association between pain and race/ethnicity, relaying socioeconomic status as the only significant predictor of a higher prevalence [44]. This is consistent with other socioeconomic inequalities, as individuals in a socioeconomic disadvantage are more likely to develop chronic pain conditions compared to affluent individuals [45,46,47].

### 4.3. Balance and CPM

Our results showed patients with better balance had more effective CPM. Balance involves complex intersensory communication in a continuous feedback loop between sensory and motor functions [48]. Pain is thought to interfere with this physiological process and worsen the ability to maintain posture control. This is observed in a meta-analysis showing a significant decrease in multicomponent balance in participants with pain [49]. It also suggests increased duration and perception of pain interfering with balance, possibly due to physiological alterations of higher cognitive areas in the central sensitization theory. Congruent results are observed in the literature for the OA population: a systematic review demonstrated that individuals with knee OA had, in a variety of scales, worsened balance [50].

### 4.4. Brain Oscillatory Correlates of CPM

We found dysfunctional CPM associated with higher delta relative power in the frontal and central regions. A few studies have assessed the oscillatory patterns associated with CPM in patients with chronic pain [21,22,23,51]. Although there are some contradictory findings, most studies have reported alterations in low-frequency bands such as delta and theta oscillations [21,23,51]. Delta frequencies are generated in the anterior medial frontal cortex and subcortical nuclei like the nucleus accumbens and PAG [52]. These are important regions for pain control and the activation of the DPMS [53]. Mainly, delta oscillatory activity has been associated with compromised neuronal function and cognitive processes [54,55]. Frontocentral delta and theta oscillatory activity could be surrogates of DPMS activation. For instance, Goudman et al. [51] reported predominant delta event-related potentials in healthy subjects, corresponding to top-down nociceptive inhibitory mechanisms attributed to the involvement of the PAG. Thus, we hypothesize that altered oscillatory activity in these bands could represent compromised pain inhibitory control in OA. This is supported by our previous studies in patients with fibromyalgia and neuropathic pain post spinal cord injury, where we reported an association between CPM and higher theta and less event-related desynchronization in the delta band [21,23]. Also, an association between delta oscillatory activity and intracortical inhibition in chronic pain has been reported, suggesting a role of these oscillations in the central sensitization process [56].

### 4.5. Negative Findings

There is evidence in the literature that age, sex, and emotional outcomes are related with the CPM response [20], which we did not observe. One explanation could be the characteristics of our sample: the majority were women (70%) with similar ages and depression/anxiety scores. This creates a homogeneous sample, thus decreasing the comparison power.

## 5. Limitations

Considering its exploratory nature, our study has some limitations. First, the absence of a control group in our study prevents us from assuming that the associations found can also be present in patients with OA without pain or healthy subjects. Moreover, we did not perform corrections for multiple analyses, which could have led to an increase in type-two errors in our results. Nonetheless, although our sample size was powered enough to detect the significant association of up to 10 covariates [24], given this is an exploratory study, our results require further confirmation.

## 6. Conclusions

Our results show worse outcomes in functional scales (such as activity-related pain (WOMAC) and BBS) and higher delta waves in the frontal and central areas of the brain associated with a less efficient CPM. Moreover, race can modify the association between pain intensity and CPM. These results suggest interesting insights of a potential modulation of central nervous areas in CPM, opening the avenue for further exploration. Longitudinal analyses could explore how pain-processing mechanisms are associated with response to treatment, allowing for individualized therapies to be used in clinical practice.

## Figures and Tables

**Table 1:** Demographics and clinical characteristics.

**Demographics**	
Age	68.46 ± 9.84
Sex (%)	
Male	15 (18)
Female	70 (82)
Race (%)	
White	53 (62)
Black	11 (13)
Mixed	17 (20)
Asian	4 (5)
Education Level (%)	
Illiterate	2 (2)
Elementary	34 (40)
High School	26 (31)
Superior	23 (27)
BMI	32.26 ± 5.31
Most affected side of the knee (%)	
Left	5 (6)
Right	16 (20)
Bilateral	59 (74)
**Clinical characteristics**	
Time of ongoing pain (months)	97.4819 ± 102.94
Pain Catastrophizing	13.83 ± 10.89
WOMAC Pain Score	10.42 ± 4.02
Average of VAS Pain	5.37 ± 2.14
HAM-D	9.08 ± 5.61
HAD-Anxiety	5.71 ± 4.09
MOCA	21.66 ± 5.00
Sf36	55.55 ± 20

BMI: Body Mass Index, WOMAC Pain: Western Ontario and MacMaster Universities Osteoarthritis Index Pain section, VAS Pain: Visual analogue Scale for Pain, HAM-D: Hamilton Depression Rating Scale, HAD-Anxiety: Hospital Anxiety and Depression Scale—Anxiety section, MOCA: Montreal cognitive assessment, SF36: 36-Item Short Form Survey.

**Table 2:** Model of multivariate analysis with active-related pain associated with CPM in both knees.

Adjusted R-Squared: 0.09			
Baseline Variables	β-Coefficient	*p*-Value	95% CI
WOMAC Pain Score	−0.13	0.003 *	−0.21 to −0.04
Pain catastrophizing scale	0.005	0.721	−0.02 to 0.03
Race	0.09	0.773	−0.53 to 0.72
Age	−0.02	0.212	−0.05 to 0.01
Sex	0.25	0.481	−0.45 to 0.96

WOMAC Pain Score: Western Ontario and MacMaster Universities Osteoarthritis Index Score Pain section. * *p* < 0.05.

**Table 3:** Model of multivariate analysis with significant functional variables and pain associated with CPM in both knees.

Adjusted R-Squared: 0.11			
Baseline Variables	β-Coefficient	*p*-Value	95% CI
WOMAC Pain Score	−0.08	0.014 *	−0.156 to −0.018
Berg Balance Scale	0.04	0.033 *	0.003 to 0.080
Race	0.15	0.592	−0.423 to 0.739
Sex	0.22	0.511	−0.472 to 0.927

* *p* < 0.05.

**Table 4:** Model of multivariate analysis with significant neurophysiological variables associated with CPM in both knees.

EEG Variables	β-Coefficient	*p*-Value	95% CI
**Frontal area**			
Adjusted R-squared: 0.08			
Relative power of delta waves	−3.11	0.021 *	−5.90 to −0.33
Pain catastrophizing scale	−0.02	0.164	−0.05 to −0.00
Race	−0.35	0.362	−1.14 to 0.43
Age	−0.003	0.843	−0.039 to 0.032
Sex	−0.25	0.652	−1.42 to 0.90
**Central area**			
Adjusted R-squared: 0.07			
Relative power of delta waves	−3.23	0.040 *	−6.34 to −0.11
Pain catastrophizing scale	−0.02	0.180	−0.05 to 0.01
Race	−0.26	0.503	−1.07 to 0.53
Age	−0.005	0.751	−0.04 to 0.03
Sex	−0.25	0.660	−1.42 to 0.92

* *p* < 0.05; EEG: Electroencephalogram.

## Data Availability

The data that support the findings of this study are available on request from the corresponding author.

## Supplementary Materials

The following supporting information can be downloaded at: https://www.mdpi.com/article/10.3390/life13081697/s1. References [24,31,32,33,34,57,58,59,60,61,62,63,64,65,66,67,68,69,70] have been cited.
