# Supplementary material for: Functional and Neural Correlates Associated with Conditioned Pain Modulation in Patients with Chronic Knee Osteoarthritis Pain: A Cross-Sectional Study
Source: Life (Basel). 2023 Aug 7;13(8):1697. doi: 10.3390/life13081697 (PMC10455308; doi:10.3390/life13081697)
Supplement: Supplementary file 1 [file life-13-01697-s001.zip › life-2487764-supplementary.pdf]

Supplementary materials

Text S1.

Questionnaires and Assessments

**Adapted from our published protocol and previous published articles related to this osteoarthritis cohort that are under review: "Deficit of inhibition as a marker of neuroplasticity - DEFINE study - in rehabilitation: a longitudinal cohort study protocol - Simis et al. 2021"(1)*

Catastrophizing variables

The Pain Catastrophizing scale is a 9 items likert scale that varies from 0-5 that correspond to the words "almost never" and "almost always" in its extremes. Higher scores reflect the presence of catastrophizing thoughts (2, 3).

Emotion related variables

Hamilton Depression Rating Scale (HAM-D): This scale assesses how the patient has been feeling in the last seven days, including the day of application. 17 items are asked to the subject, which can be scored on a Likert scale ranging from 0 to 2 or 0 to 4, depending on the intensity of the symptom. The total number of points varies between 0 and 52 points. To verify the presence of depression, the scores must add up to at least 8 points in the original version (4)

Hospital Anxiety and Depression Scale (HADS): A 14-item scale that quantifies and qualifies symptoms of anxiety and depression, asking multiple choice questions, based on how the subject felt during the last week. It consists of two subscales, one for anxiety and another for depression, with seven items each. The global score in each subscale ranges from 0 to 21. Its objective is to detect mild degrees of affective disorders in non-psychiatric environments (5).

Cognitive variables

Montreal Cognitive Assessment (MOCA): Assess the patient's dominance over the following domains: their cognitive function's executive function, visuo-spacial ability, memory, attention, concentration, occupational memory, language, and temporal and spatial orientation. The scale has a maximum score of 30 points and an application time of approximately 10 minutes (6).

Sleep variables

Epworth sleepiness scale: Investigates the degree of daytime sleepiness. It is a self-applied questionnaire that evaluates the probability of falling asleep in 8 everyday situations (7).

Functionality variables

Berg Balance Scale: Consists of 13 tasks (reaching, turning, transferring, standing up, etc.) that measures the static and dynamic balance components of an individual. Each item ranges from 0-4, higher scores are related to better ability to perform the task. The maximum score is 56 points (8).

10-meter walking test: Evaluates a patient's short-distance walk speed. It is recommended that the subject walks 14 meters so that the 2 initial and final meters be disregarded. The subject is asked to walk at their normal speed (9).

6-minute walking test: Assess the maximum distance a subject can walk on a plane, rigid surface in six minutes through a 30-meter track. It is recommended that this be a 30-meter walk, lapped every 3 meters in which turning points are set with a cone (9).

Timed Up and Go (TUG): This test assesses an individual's mobility level, measuring the amount of time it takes for the subject to stand up from a chair without using their arms, walk a 3-meter distance, turn 180° and get back to sit on the chair (10).

General functionality

Medical Outcomes Short-Form Health Survey (SF-36): Is a survey that assess the overall health of the individual. It consists of 36 questions related to eight components, including functional capacity, physical aspects, pain, general health status, vitality, social aspects, emotional aspects, and mental health. The last question compares between the subject's current health and that of a year ago (11).

Intrinsic variables of the disease

Kellgren-Lawrence Radiographic Classification of OA: Is a classifying method of the severity of knee osteoarthritis (OA), which divides it in five degrees: 0 (without osteoarthritis) to 4 (large osteophyte, marked narrowing of the joint space, severe sclerosis, and definite deformity of bony extremities) (12).

Neurophysiological variables

Resting-state electroencephalography (EEG)

EEG acquisition

We recorded the EEG following a standardized approach (13) in a quiet room. Assessors asked the patients to sit comfortably, have their sight directed naturally below the horizon line, not move, or talk, and relax as much as possible. The investigator made sure they did not fall asleep by observing the patient and verbally calling his attention if drowsiness was noticed. We recorded the resting-state EEG for 5 minutes with eyes closed using a 128-channel EGI system (Electrical Geodesics, Inc) (EGI, Eugene, USA). The EEG was recorded with a band-pass filter of 0.3–200 Hz and digitized at the sampling rate of 250 Hz.

Resting-state spectral power analysis

We exported the data for offline analysis with EEGLab (14) and MATLAB (MATLAB R2012a, The MathWorks Inc. Natick, MA, 2000). EEG was re-referenced to the average; we used finite impulse response filters, one high-pass filter of 1 Hz and a low-pass filter of 50 Hz, followed by manual artifact detection and rejection by a blinded assessor to exclude the existence of any signal of drowsiness (attenuation of the alpha rhythm), epileptiform or any abnormal discharges prior to admission into the full study (no epileptiform or abnormal discharges were found). After, a manual artifact detection and rejection and Independent Component Analysis (ICA) was performed; finally, we removed the ICs associated with artifacts and reconstructed the signal. (15) We processed the artifact-free data using pop_spectopo EEGLab function with Fast Fourier Transformation with 5s windows with 50% overlap. We calculated absolute power (µV2) and relative power (power in a specific frequency range/total power from 1 to 40Hz) for the following frequency bands: delta (1–4 Hz), theta (4–8 Hz), alpha (8–13 Hz), beta (13-30 Hz), and the sub-bands: low beta (13–20 Hz) and high beta (20–30 Hz). We calculated all the EEG-related measurements from three regions of interest (ROIs): the central, parietal, and frontal areas, since they are important cortical regions involved in pain perception. (16) Also, we selected and averaged the electrodes representing these regions.

Transcranial magnetic stimulation (TMS)

We used the Magstim Rapid® stimulator (The Magstim Company Limited, UK) to assess the TMS measurements. A 70mm coil in figure-of-eight at 45 degrees of the scalp was placed to send a perpendicular pulse over the right and left motor cortex (for all assessments). The assessor managed the coil stability and direction without neuronavigation. We recorded the muscular response to the stimulus using surface electromyography (EMG) with Ag/AgCl electrodes positioned on the first dorsal interosseous (FDI) muscle of the hand and the grounding electrode positioned on the wrist (17).

We performed a bilateral upper limb assessment and used anatomical references for motor cortex localization. Initially, we identified the vertex and then, we made a mark 5 cm from the vertex towards the ear tragus in the coronal plane. We determined the hotspot as the location with the highest and most stable motor evoked potential (MEP) amplitudes over the FDI. The resting motor threshold (rMT) was defined as the minimum intensity necessary for a single TMS pulse on the hot spot to generate an MEP, with at least 50µV peak to peak amplitude, in 50% of attempts (18). We performed the following measures: MEP (intensity at 120% of rMT, we calculated the peak-to-peak amplitude), cortical silent period (CSP), which represents the temporary suppression of electromyographic activity during a sustained voluntary contraction.

We also performed paired-pulse protocols of intracortical inhibition (SICI), assessed by interstimulus intervals of 2 ms; and intracortical facilitation (ICF) assessed by 10 ms interim stimulus intervals (18). Ten randomized stimuli were applied at each interval and the average were calculated.

For the TMS neurophysiological measurements, we pooled the rMT, CSP, SICI, ICF, and MEP results from each hemisphere to obtain a bi-hemispheric average. This approach can be justified due to the bi-hemispheric nature of pain perception (19); TMS data were recorded and stored in a computer for offline analysis.

Table S1. Linear Univariate Analysis with demographics, clinical, genetical and neurophysiological variables associated with CPM in both knees.

Baseline Variable	β-coefficient	P value
Time of ongoing pain	.0017792	0.200
Weight	.0115424	0.224
Height	3.110952	0.038*
Education	. 2080472	0.206
Pain catastrophizing	-.0212983	0.105
SF-36 Physical function section	.0080106	0.199
SF-36 Pain section	.008469	0.187
10-meter walking test	-.0542442	0.018*
6-meter walking test	.003503	0.006*
Timed Up and Go	-.0415621	0.026*
Berg Balance Scale	. 053779	0.005*
WOMAC Pain Score	-.1016224	0.004*
WOMAC rigidity score	-.0986124	0.161
WOMAC functionality	-.0279553	0.007*
WOMAC total score	-.021881	0.005*
VAS in both knees (average)	-.1185421	0.071
Motor threshold average from both hemispheres at baseline	.017958	0.137
WOMAC Pain score categorized by a cut off of 50% of improvement	-.7823941	0.005*
VAS in both knees (average) categorized by a cut off of 50% of improvement	-.6036078	0.034*
Relative power of delta waves in the frontal area	-2.647974	0.052
Relative power of delta waves in the central area	-2.99604	0.049*
Relative power of alpha waves in the central area	1.802287	0.161
Relative power of high alpha waves in the central area	3.595126	0.195
Ratio of relative power of alpha and theta waves in the central area	.23927	0.116
Relative power of delta waves in the parietal area	-2.522748	0.079
Relative power of high alpha waves in the parietal area	2.657956	0.186
Ratio of Relative power of alpha and theta waves in the parietal area	.1468904	0.080
Relative power of delta waves in the occipital area	-2.395194	0.085
SF-36 intensity	.0088563	0.171
Polymorphism BDNF	.4276138	0.227

* Values with a $p<0.05$

Table S2. Logistic Univariate Analysis with demographics, clinical, genetical and neurophysiological variables associated with CPM in both knees.

Baselines Variable	OR	P value
Sex	2.065934	0.205
Education	1.549256	0.102
Treatment duration	.9950589	0.199
MOCA	1.081678	0.086
SF-36 Pain section	1.017672	0.103
10-meter walking test	.9411463	0.141
6-meter walking test	1.004205	0.052
Timed Up and Go	.9448089	0.112
Berg Balance Scale	1.076454	0.032*
WOMAC Pain score	.8802669	0.032*
WOMAC Rigidity score	.7704236	0.033*
WOMAC functionality score	.9556753	0.012*
WOMAC Total Score	.9654784	0.010*
VAS in both knees (average)	.7764289	0.024*
Motor threshold average from both hemispheres at baseline	1.022538	0.257
WOMAC Pain score categorized by a cut off of 50% of improvement	.3471075	0.020
VAS in both knees (average) categorized by a cut off of 50% of improvement	.2692308	0.005
SF-36_intensity	1.018622	0.094
SF-36_interference	1.009431	0.230
BDNF	1.931486	0.212
Polymorphism BDNF	1.888889	0.247

* Values with a $p<0.05$

Table S3. Model of Interaction between active-related pain and CPM in both knees

			Adjusted R-squared: 0.13
**Baseline variables**	**β-coefficient**	**p-value**	**95% CI**
**WOMAC Pain Score**	-0.07	0.12	-0.17 to 0.02
**Pain catastrophizing scale**	0.005	0.70	-0.02 to 0.03
**Race**	1.78	0.03	0.12 to 3.44
**Age**	-0.01	0.22	-0.05 to 0.01
**Sex**	0.20	0.55	-0.49 to 0.90
Interaction			
**WOMAC pain score and Race**	-0.15	0.03*	-0.29 to -0.01

Table S4. Model of multivariate analysis with active-related pain associated with CPM in both knees categorized by percentage change

Baseline variables	OR	p-value	95% CI
**WOMAC Pain Score**	0.79	0.008*	0.67 to 0.94
**Pain catastrophizing scale**	1.02	0.31	0.97 to 1.08
**Race**	0.51	0.24	0.16 to 1.58
**Age**	0.95	0.10	0.93 to 1.01
**Sex**	3.39	0.06	0.93 to 12.32

Table S5. Model of multivariate analysis with the Visual Analogue Score for Pain associated with CPM in both knees categorized by percentage change

Baseline variables	OR	p-value	95% CI
**Bilateral Visual Analogue Scale of Pain**	0.73	0.02*	0.56 to 0.96
**Pain catastrophizing scale**	1.01	0.68	0.96 to 1.06
**Race**	0.48	0.20	0.15 to 1.48
**Age**	0.98	0.58	0.93 to 1.03
**Sex**	2.82	0.09	0.84 to 9.45

Table S6. Model of multivariate analysis with active-related pain associated with CPM in both knees categorized by zero cut-off

Baseline variables	OR	p-value	95% CI
**WOMAC Pain Score**	0.73	0.007*	0.58 to 0.91
**Pain catastrophizing scale**	1.03	0.35	0.96 to 1.10
**Race**	0.45	0.27	0.11 to 1.83
**Age**	0.95	0.23	0.88 to 1.03
**Sex**	2.52	0.22	0.56 to 11.30

Table S7. Model of multivariate analysis with the Visual Analogue Score for Pain associated with CPM in both knees categorized by zero cut-off

Baseline variables	OR	p-value	95% CI
**Bilateral Visual Analogue Scale of Pain**	0.68	0.02*	0.49 to 0.95
**Pain catastrophizing scale**	1.00	0.90	0.94 to 1.06
**Race**	0.45	0.25	0.11 to 1.74
**Age**	0.99	0.89	0.93 to 1.06
**Sex**	2.17	0.28	0.52 to 9.01

References

1. Simis M, Imamura M, Sampaio de Melo P, Marduy A, Battistella L, Fregni F. Deficit of Inhibition as a Marker of Neuroplasticity (DEFINE Study) in Rehabilitation: A Longitudinal Cohort Study Protocol. Frontiers in neurology. 2021;12:695406-.
2. Sardá Junior J NM, Pereira IA, Pimenta CA de M, Asghari A, Cruz RM. Validação da Escala de Pensamentos Catastróficos sobre Dor. Acta Fisiátrica. 2008;15.
3. Sullivan MJL, Bishop SR, Pivik J. The Pain Catastrophizing Scale: Development and validation. Psychological Assessment. 1995;7(4):524-32.
4. Freire M, Figueiredo V, Vasconcelos A, Jansen K, Silva R, Magalhães P, et al. Escala Hamilton: estudo das características psicométricas em uma amostra do sul do Brasil. Jornal Brasileiro de Psiquiatria. 2014;63:281-9.
5. Botega NJ, Bio MR, Zomignani MA, Garcia C, Jr., Pereira WA. [Mood disorders among inpatients in ambulatory and validation of the anxiety and depression scale HAD]. Revista de saude publica. 1995;29(5):355-63.
6. Freitas S, Simões MR, Martins C, Vilar M, Santana I. Estudos de adaptação do Montreal Cognitive Assessment (MoCA) para a população portuguesa %J Avaliação Psicológica. 2010;9:345-57.
7. Johns MW. Reliability and factor analysis of the Epworth Sleepiness Scale. Sleep. 1992;15(4):376-81.
8. Stevenson TJ. Detecting change in patients with stroke using the Berg Balance Scale. The Australian journal of physiotherapy. 2001;47(1):29-38.
9. Steele B. Timed walking tests of exercise capacity in chronic cardiopulmonary illness. Journal of cardiopulmonary rehabilitation. 1996;16(1):25-33.
10. Podsiadlo D, Richardson S. The timed "Up & Go": a test of basic functional mobility for frail elderly persons. Journal of the American Geriatrics Society. 1991;39(2):142-8.
11. Ferraz CRJRBR. Santos W, Meinão I, Quaresma MR. Tradução para a língua portuguesa e validação do questionário genérico de avaliação de qualidade de vida SF-36 (Brasil SF-36). 1999;39:143-50.
12. Kellgren JH, Lawrence JS. Radiological assessment of osteo-arthrosis. Annals of the rheumatic diseases. 1957;16(4):494-502.

13. Nuwer MR, Lehmann D, da Silva FL, Matsuoka S, Sutherling W, Vibert JF. IFCN guidelines for topographic and frequency analysis of EEGs and EPs. The International Federation of Clinical Neurophysiology. Electroencephalography and clinical neurophysiology Supplement. 1999;52:15-20.
14. Delorme A, Makeig S. EEGLAB: an open source toolbox for analysis of single-trial EEG dynamics including independent component analysis. Journal of neuroscience methods. 2004;134(1):9-21.
15. Delorme A, Sejnowski T, Makeig S. Enhanced detection of artifacts in EEG data using higher-order statistics and independent component analysis. Neuroimage. 2007;34(4):1443-9.
16. Jensen KB, Regenbogen C, Ohse MC, Frasnelli J, Freiherr J, Lundström JN. Brain activations during pain: a neuroimaging meta-analysis of patients with pain and healthy controls. Pain. 2016;157(6):1279-86.
17. Malcolm MP, Triggs WJ, Light KE, Shechtman O, Khandekar G, Gonzalez Rothi LJ. Reliability of motor cortex transcranial magnetic stimulation in four muscle representations. Clinical neurophysiology : official journal of the International Federation of Clinical Neurophysiology. 2006;117(5):1037-46.
18. Rossini PM, Burke D, Chen R, Cohen LG, Daskalakis Z, Di Iorio R, et al. Non-invasive electrical and magnetic stimulation of the brain, spinal cord, roots and peripheral nerves: Basic principles and procedures for routine clinical and research application. An updated report from an I.F.C.N. Committee. Clinical neurophysiology : official journal of the International Federation of Clinical Neurophysiology. 2015;126(6):1071-107.
19. Schwenkreis P, Janssen F, Rommel O, Pleger B, Völker B, Hosbach I, et al. Bilateral motor cortex disinhibition in complex regional pain syndrome (CRPS) type I of the hand. Neurology. 2003;61(4):515-9.
